# Agricultural chemicals: life changer for mosquito vectors in agricultural landscapes?

**DOI:** 10.1186/s13071-016-1788-7

**Published:** 2016-09-13

**Authors:** Tabitha W. Kibuthu, Sammy M. Njenga, Amos K. Mbugua, Ephantus J. Muturi

**Affiliations:** 1 Institute of Tropical Medicine, Jomo Kenyatta University of Agriculture and Technology, Nairobi, Kenya; 2 Eastern and Southern Africa Centre of International Parasite Control, Kenya Medical Research Institute, Nairobi, Kenya; 3 College of Health Sciences, Jomo Kenyatta University of Agriculture and Technology, Nairobi, Kenya; 4 Illinois Natural History Survey, Prairie Research Institute, University of Illinois, Champaign, USA; 5 Present Address: U.S.D.A., Agricultural Research Service, National Center for Agricultural Utilization Research, Crop Bioprotection Research Unit, 1815 N. University St., Peoria, IL 61604 USA

**Keywords:** Agricultural chemicals, Sublethal concentrations, Life history traits, *Anopheles arabiensis*, *Culex quinquefasciatus*

## Abstract

**Background:**

Although many mosquito species develop within agricultural landscapes where they are potentially exposed to agricultural chemicals (fertilizers and pesticides), the effects of these chemicals on mosquito biology remain poorly understood. This study investigated the effects of sublethal concentrations of four agricultural chemicals on the life history traits of *Anopheles arabiensis* and *Culex quinquefasciatus* mosquitoes.

**Methods:**

Field and laboratory experiments were conducted to examine how sublethal concentrations of four agricultural chemicals: an insecticide (cypermethrin), a herbicide (glyphosate), and two nitrogenous fertilizers (ammonium sulfate and diammonium phosphate) alter oviposition site selection, emergence rates, development time, adult body size, and longevity of *An. arabiensis* and *Cx. quinquefasciatus*.

**Results:**

Both mosquito species had preference to oviposit in fertilizer treatments relative to pesticide treatments. Emergence rates for *An. arabiensis* were significantly higher in the control and ammonium sulfate treatments compared to cypermethrin treatment, while emergence rates for *Cx. quinquefasciatus* were significantly higher in the diammonium phosphate treatment compared to glyphosate and cypermethrin treatments. For both mosquito species, individuals from the ammonium sulfate and diammonium phosphate treatments took significantly longer time to develop compared to those from cypermethrin and glyphosate treatments. Although not always significant, males and females of both mosquito species tended to be smaller in the ammonium sulfate and diammonium phosphate treatments compared to cypermethrin and glyphosate treatments. There was no significant effect of the agrochemical treatments on the longevity of either mosquito species.

**Conclusions:**

These results demonstrate that the widespread use of agricultural chemicals to enhance crop production can have unexpected effects on the spatial distribution and abundance of mosquito vectors of malaria and lymphatic filariasis.

## Background

Mosquitoes transmit some of the most devastating infectious diseases including malaria, lymphatic filariasis (LF), and dengue. Transmission of these diseases is largely influenced by mosquito distribution, abundance and fitness, which are in turn dependent on the quality of aquatic habitats where egg hatch and larval development occurs [1, 2]. Because mosquitoes do not provide parental care to their offspring, natural selection should favor the ability of gravid females to select aquatic habitats that maximize egg hatch and offspring fitness [3]. This process requires complex integration of biological, chemical and physical cues by gravid females [4]. Chemical contaminants can potentially disrupt this process by modifying the quality and attractiveness of the aquatic habitats and vector biologists are faced with the challenge of determining the impact of these chemicals on mosquito ecology, behavior, and ability to transmit pathogens.

Agrochemicals (fertilizers and pesticides) are one of the major classes of chemical contaminants that can potentially affect mosquito oviposition behavior and offspring fitness. Every year, an estimated 2.4 million tons of pesticides [5] and 180.1 million tons of fertilizers [6] are used worldwide to improve agricultural production and human health. The extensive use of these chemicals has led to their recurrent detection in many surface waters [7, 8] increasing the potential for aquatic communities to be exposed to these chemicals. Immature stages (larvae and pupae) of many mosquito species including the vectors of malaria and LF develop in a variety of ephemeral and permanent water bodies situated within agricultural areas where they are potentially exposed to agrochemicals that are either applied to these farmlands or transported from nearby farmlands through spray drift, surface runoff, and/or leaching. However, despite the notable potential for mosquitoes to be exposed to agrochemicals, the implications of agrochemical use on the ecology and behavior of major vectors remain poorly understood.

Agricultural application of nitrogenous fertilizers is a major source of nitrate contamination of aquatic systems. In many countries, nitrate concentration in surface and ground water ranges from 5 mg/l to > 100 mg/l, and due to its high solubility in water, nitrate has high mobility in the environment [7]. High levels of nitrates may promote mosquito production by enhancing proliferation of algal blooms and other microbial assemblages that serve as food for mosquito larvae [9, 10]. Fertilizer application also may alter the physical and chemical properties of the aquatic habitats making them attractive oviposition sites for mosquitoes [11–13]. This may explain why fertilizer application in rice fields is often associated with a dramatic increase in mosquito larval populations [10–12]. However, experimental studies to decipher the impact of nitrogenous fertilizers on epidemiologically relevant mosquito life history traits in the absence of other confounding factors are lacking.

Pesticides may also affect mosquito populations and communities both directly and indirectly. When initially applied, pesticides have lethal effects but can break down over time and switch from being lethal to sublethal and eventually to having no effects [14]. A shift in pesticide concentrations from lethal to sublethal is clearly demonstrated by the rapid reduction in mosquito larvae and their predators after pesticide application, followed by the resurgence of mosquito populations thereafter since they recover faster than their predators [15, 16]. Lethal concentrations of pesticide may produce compensatory effects by killing a fraction of the population and releasing the survivors from larval competition [17–19]. Conversely, insights from other aquatic insects and amphibians suggest that sublethal concentrations of insecticides can cause morphological, behavioral, and physiological impairments [20–24] that can become more deleterious when presented in the presence of other environmental stressors [24–26]*.* Similar effects have been reported in *Culex* and *Aedes* mosquitoes where larval exposure to certain pesticides alters their emergence rates, development time, longevity, body size, sex ratio, and vector competence [18, 27–30]. In addition, C*ulex* mosquitoes preferred to oviposit in carbaryl-contaminated pools while *Anopheles* mosquitoes had no preference for either control or carbaryl-contaminated treatments [31].

We conducted a series of field and laboratory experiments to determine how *Anopheles arabiensis* and *Culex quinquefasciatus* mosquitoes respond to two commonly used pesticides (α- cypermethrin and glyphosate) and two commonly used fertilizers (ammonium sulfate and diammonium phosphate). We examined this by quantifying several life history traits including oviposition site selection, development time, emergence rates, adult body size and longevity. *Culex quinquefasciatus* is a major worldwide vector of LF [32–34] while *An. arabiensis* is an important vector of both malaria and LF and has become the most dominant malaria vector in areas where insecticide-treated bed nets and indoor residual spraying have been implemented [35–37]. We tested the hypotheses that: (i) the fertilizer and pesticide treatments alter oviposition site selection by the two mosquito species; and (ii) agrochemical treatments would differentially affect the development time, emergence rates, adult body size and longevity of the two mosquito species.

## Methods

### Study area

This study was conducted at the Mwea Irrigation and Agricultural Development (MIAD) experimental station and at Kangichiri and Kariua villages in the Mwea rice irrigation scheme, 100 km north-east of Nairobi, in Mwea Division, Kenya. The two villages were selected based on the presence of large populations of *An. arabiensis* and *Cx. quinquefasciatus* and proximity to MIAD. Mwea occupies the lower altitude zone of Kirinyaga County in an expansive low-lying area mainly characterized by black cotton soils. The mean annual rainfall is 950 mm with maximum amount falling in April-May (long rains) and October-November (short rains). The average maximum temperatures are in the range of 16–26.5 °C. Relative humidity varies from 52 to 67 %. According to the 2009 national census, Mwea Division has approximately 150,000 persons in 25,000 households. The Mwea Rice Irrigation Scheme is located in the west central region of Mwea Division and covers an area of about 13,640 ha. More than 50 % of the Scheme area is used for rice cultivation. The remaining area is used for subsistence farming, grazing and community activities. *Anopheles arabiensis* is the dominant vector of malaria in Mwea, and the only sibling species of the *An. gambiae* species complex recorded in the area [38, 39]. *Culex quinquefasciatus* is the most abundant species of *Culex* in the area [40].

### Agrochemicals

Alpha cypermethrin is a pyrethroid insecticide applied as an ultra-low volume spray to control insects in both large-scale commercial agriculture and small-scale agricultural settings. Pyrethroids are increasingly replacing organophosphates and carbamates in agriculture because of their effectiveness, lower application rates, and lower toxicity to mammals [41]. Up to 3,114 μg/l of permethrin have been observed in water bodies [42].

The herbicide glyphosate is of particular interest to understanding the consequences of pesticide use on pathogen transmission because of its widespread and abundant use [43]. By volume, it is one of the most widely used herbicides and is commonly used for agriculture, horticulture, viticulture and silviculture purposes, as well as garden maintenance [44]. Glyphosate is absorbed through foliage and minimally through roots and translocated throughout the plant [45]. Its primary action is blocking an enzyme that plants need to make aromatic amino acids and proteins thus killing the plants within days [46]. Worldwide, around 650,000 tons of glyphosate products were used in 2011 [47]. Glyphosate use has continued to increase largely due to the production of genetically modified crops and is expected to double by 2017 [48]. The maximum concentration of glyphosate observed in water bodies is 5,200 μg/l [49].

Ammonium sulfate [(NH_4_)_2_SO_4_] and diammonium phosphate [(NH_4_)_2_HPO_4_] are inorganic fertilizers that are commonly used to supplement the soil with three basic elements that are essential for plant growth nitrogen, sulfur and phosphorus. The annual world demand for nitrogen and phosphate fertilizers stands at 113.1 and 42.7 million tons, respectively and is expected to increase over the next two years [50]. These nutrients are important sources of ground and surface water pollution. Nitrate concentrations in water bodies near intensively cultivated and fertilized areas can be greater than 100 mg/l [51] while that of phosphate can be as high as 9.45 mg/l [52].

### Laboratory studies on the effect of agrochemicals on oviposition site selection by *An. arabiensis* and *Cx. quinquefasciatus*

Blood-engorged females of *An. arabiensis* and *Cx. quinquefasciatus* were collected inside human dwellings in Kangichiri and Kariua villages using manual aspirators. At MIAD, 20 randomly selected females of either species were transferred into one of 9 (*An. arabiensis*) and 15 (*Cx. quinquefasciatus*) insect rearing cages (30 × 30 × 30 cm) and provided continuous access to 10 % sucrose. For *Cx. quinquefasciatus* oviposition, each cage was provisioned with five Petri dishes containing either 50 ml of tap water (control) or 50 ml of agrochemical-spiked water generating four treatments with a final concentration of 0.1 mg/l α-cypermethrin, 0.5 mg/l glyphosate, 845 mg/l ammonium sulfate, or 845 mg/l diammonium phosphate. These pesticide concentrations are within the range that is commonly found in aquatic habitats [42, 49, 51, 52]. In contrast, the fertilizer concentrations used in this study are much higher than observed in nature. Because fertilizer application in rice fields within the study sites and other parts of Africa is done manually through broadcasting, it is expected that some parts of the rice fields receive high doses of fertilizers similar to those used in this study. Therefore we sought to determine how potentially higher doses of fertilizers may affect mosquito ecology. A similar setup was used for *An. arabiensis* except that the Petri dishes were lined with filter papers moistened with respective agrochemical treatments. A single Petri dish was placed on each corner of a cage and the fifth one was placed in the middle of the cage. The treatments were rotated daily to eliminate positional bias. The number of eggs laid in each Petri dish were counted and recorded every day for 31 days and their sums computed. Agrochemical-treated filter papers were replaced each day.

### Field studies on the effect of agrochemicals on oviposition site selection by *Culex quinquefasciatus*

This experiment was conducted between 1st August, 2014 and 20th September, 2014 under field conditions since *Culex* egg rafts are easy to monitor compared to *Anopheles* eggs that are laid individually. The experiment was conducted in five randomly selected homesteads in Kangichiri village. In each homestead, five 20-litre plastic buckets containing either 3 l of fermented grass infusion (control) or 3 l of fermented grass infusion spiked with one of four agrochemicals for a final concentration of 1 mg/l α-cypermethrin, 2 mg/l glyphosate, 845 mg/l ammonium sulfate, or 845 mg/l diammonium phosphate served as the artificial oviposition sites. Because pesticides are expected to breakdown rapidly under field conditions, we deliberately used higher concentrations of α-cypermethrin and glyphosate than those used in laboratory oviposition experiment described above. Each bucket had a top lid and large openings on their upper halves to facilitate access by mosquitoes. The infusion was prepared by mixing 1 kg of fresh grass with 100 l of water and leaving it to ferment for 5 days. The homesteads were at least 60 m apart and the buckets within a homestead were 2 m apart. The buckets within each homestead were rotated daily to eliminate spatial effects. Every day, egg rafts were collected, counted, and transported to the laboratory in Petri dishes lined with moist filter papers. The infusion was replaced every three days and 13 oviposition trials were conducted.

### Effect of agrochemicals on survival and development of *An. arabiensis* and *Cx. quinquefasciatus*

Twenty first instar larvae (24 h old) of either *An. arabiensis* or *Cx. quinquefasciatus* obtained by hatching eggs from control treatments of oviposition experiments were added into tripour beakers containing either 350 ml of tap water (control) or 350 ml of tap water spiked with one of four agrochemical treatments at the following final concentrations: 0.0004 mg/l α-cypermethrin, 0.05 mg/l glyphosate, 845 mg/l ammonium sulfate, or 845 mg/l diammonium phosphate. These low α-cypermethrin and glyphosate concentrations were chosen based on concentrations observed in aquatic systems as well as in previous studies on the impact of sublethal doses of pesticides on aquatic communities. Also, our preliminary studies revealed that higher doses of α-cypermethrin were lethal to mosquito larvae (data not shown). Each treatment was replicated 6 times for a total 60 containers. The larvae were replenished with 0.05 g ground Tetramin® baby fish food once per week. Containers were examined daily until all individuals had either pupated or died. Pupae were placed in plastic vials with a small volume of water until eclosion. The date and sex of newly eclosed adults in each replicate were recorded. The adults were held individually in 75 × 20 mm plastic cups covered by nylon net and provided continuous access to 10 % sucrose solution. All cages were maintained at approximately 25–27 °C, with a relative humidity (RH) of 65–75 % and a 12:12 Light: Dark cycle. Each individual adult mosquito was monitored daily until death. Dead adults (both males and females) were preserved in plastic vials and transported to the Eastern and Southern Africa Centre of International Parasite Control (ESACIPAC), Kenya Medical Research Institute (KEMRI) where their wings were removed and mounted on microscope slides. The wings were scanned and measured from the tip (excluding the fringe) to the distal end of the allula, using VHX KEYENCE digital microscope at the Department of Entomology, Nairobi National Museum.

### Statistical analyses

Data analyses were conducted using R.3.2.5 (R Core Team) and SAS 9.4 (SAS Institute) statistical packages. Data were checked for normality and homogeneity of variances using Kolmogorov-Smirnov test. Oviposition data were log-transformed [log (x + 1)] to normalize the distribution. The means of each replicate of a treatment were compiled for each life-history trait and statistical analyses were based on these means. For each mosquito species, univariate analysis of variance (ANOVA) was used to determine the effect of agrochemical treatments on oviposition site selection, hatching rates and emergence rates (males and females combined). In the field oviposition experiment, agrochemical treatment was used as a fixed factor while trial number was used as a random factor. Multivariate analysis of variance (MANOVA) was used to determine the effect of agrochemical treatments on both male and female development time, wing length and longevity. Standardized canonical coefficients were used to describe the relative contribution of development time, wing length, and longevity to the significant treatment effects. When significant effects were obtained in both ANOVA and MANOVA tests, pair-wise differences between treatment means were compared using the Tukey-Kramer multiple comparison procedure.

## Results

### Effect of agrochemicals on oviposition site selection by *An. arabiensis* and *Cx. quinquefasciatus*

**Fig. 1:**
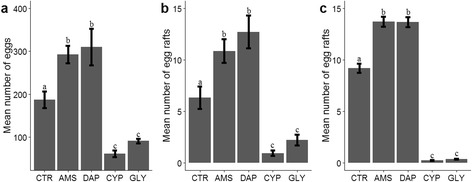
Mean number (± standard error, SE) of *An. arabiensis* eggs (**a**) and *Cx. quinquefasciatus* egg rafts (**b**, **c**) laid in different agrochemical treatments (**b** and **c** are results for laboratory and field experiments, respectively). Different lower case letters indicate significant differences between treatments. *Abbreviations*: CTR, control; AMS, ammonium sulfate; DAP, diammonium phosphate; CYP, cypermethrin; GLY, glyphosate

Agrochemical treatments had significant effects on oviposition site selection by *An. arabiensis* (*F*_(4,40)_ = 24.02, *P* < 0.001). The numbers of *An. arabiensis* eggs deposited were highest in the DAP and ammonium sulfate treatments, intermediate in the control treatment and lowest in the cypermethrin and glyphosate treatments (Fig. 1a). Similarly, results of both laboratory (*F*_(4,70)_ = 25.18, *P* < 0.001) and field experiments (*F*_(4,1245)_ = 331.37, *P* < 0.001) revealed that *Cx. quinquefasciatus* egg rafts were highest in the DAP and ammonium sulfate treatments, intermediate in the control treatment, and lowest in the cypermethrin and glyphosate treatments (Fig. 1b, c).

### Effect of agrochemicals on emergence rates, development time, wing length and longevity of *An. arabiensis* and *Cx. quinquefasciatus*

**Fig. 2:**
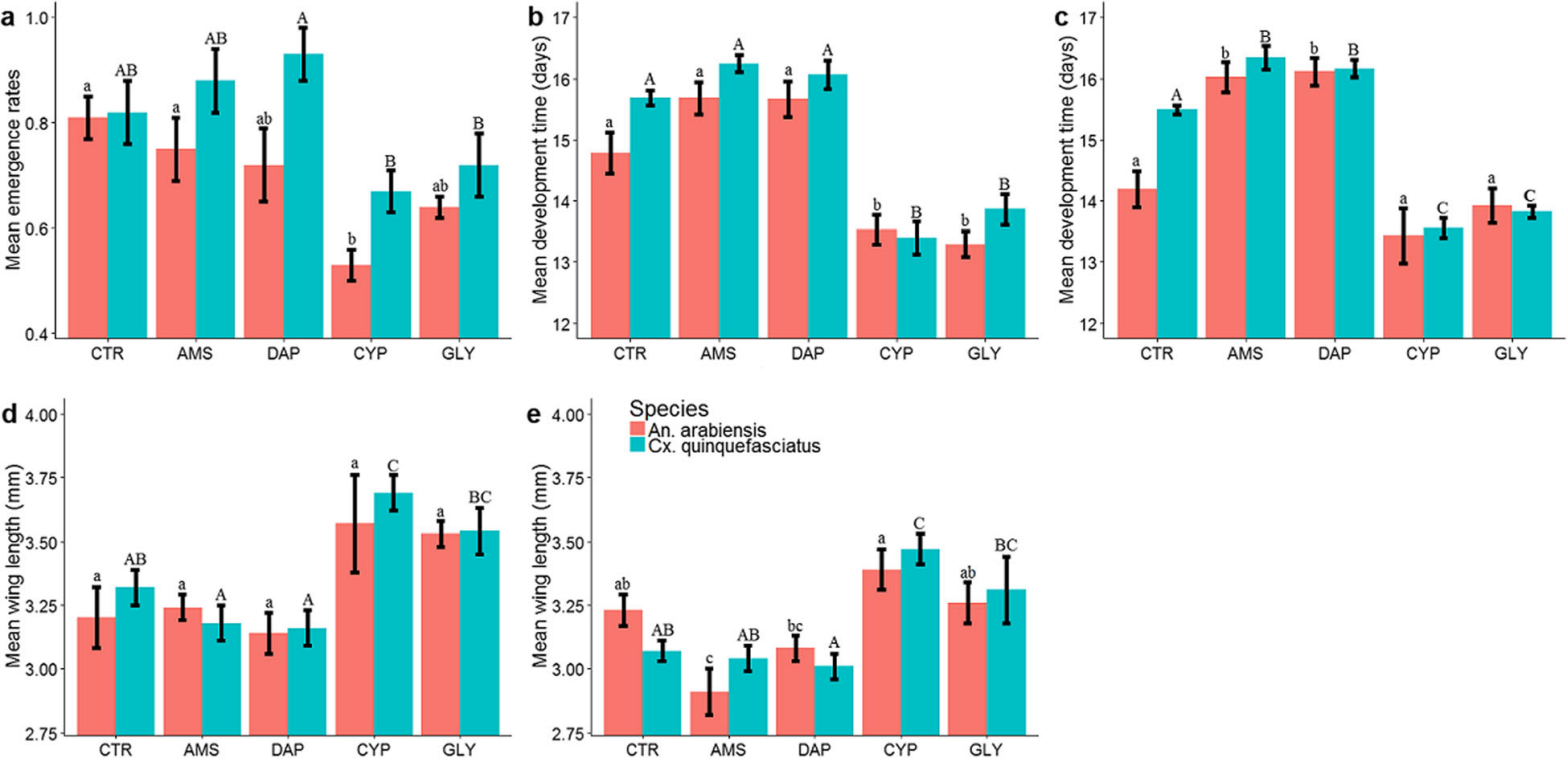
Mean emergence rates (**a**), female development time (**b**), male development time (**c**), female wing length (**d**) and male wing length (**e**) (± standard error, SE) of *An. arabiensis* and *Cx. quinquefasciatus* mosquitoes from different agrochemical treatments. Different lower and upper case letters indicate significant differences between treatments. Lower case letters are used for *An. arabiensis* and upper case letters are used for *Cx. quinquefasciatus. Abbreviations*: CTR, control; AMS, ammonium sulfate; DAP, diammonium phosphate; CYP, cypermethrin; GLY, glyphosate

Agrochemical treatments had significant effects on emergence rates of both mosquito species (*An. arabiensis*: *F*_(4,25)_ = 4.28, *P* = 0.009; *Cx. quinquefasciatus*: *F*_(4,25)_ = 4.54, *P* = 0.007). Emergence rates for *An. arabiensis* in the cypermethrin treatment were significantly lower compared to those of the control and ammonium sulfate but not glyphosate and DAP treatments (Fig. 2a). Emergence rates for *Cx. quinquefasciatus* in the cypermethrin and glyphosate treatments were significantly lower than those of DAP but not control and ammonium sulfate treatments (Fig. 2a).

For both mosquito species and sexes, multivariate analysis of variance revealed a significant effect of agrochemical treatment on development time to adulthood, wing length and longevity, with development time followed by wing length accounting for most of the variation (Table 1). *Anopheles arabiensis* females from the control, DAP, and ammonium sulfate treatments had significantly longer development times compared to those from the glyphosate and cypermethrin treatments (*F*_(4,25)_ = 17.71, *P* < 0.001; Fig. 2b). However, agrochemical treatment had no significant effect on *An. arabiensis* female wing length (*F*_(4,25)_ = 3.2, *P* = 0.30; Fig. 2d) and longevity (*F*_(4,25)_ = 1.6, *P* = 0.21). *Anopheles arabiensis* males from the DAP and ammonium sulfate treatments had significantly longer development times compared to those from the control, glyphosate and cypermethrin treatments (*F*_(4,25)_ = 16.36, *P* < 0.001; Fig. 2c). *Anopheles arabiensis* males from the ammonium sulfate treatment were significantly smaller compared to those from the control, glyphosate and cypermethrin treatments, but not from the DAP treatment (*F*_(4,25)_ = 6.32, *P* = 0.001; Fig. 2e). In addition, males from the DAP treatment were significantly smaller than those from the cypermethrin treatments (Fig. 2e). There were no significant effects of agrochemical treatment on *An. arabiensis* male longevity (*F*_(4,25)_ = 1.05, *P* = 0.40).

**Table 1:** MANOVA results on the effect of agrochemical treatments on development time to adulthood, wing length, and longevity of *An. arabiensis* and *Cx. quinquefasciatus* mosquitoes. Standardized canonical coefficients (SCC) describe the relative contribution of each response variable to significant treatment effects. Negative associations are denoted by (-)

Mosquito species	Sex	*df*	Pillai’s trace	*P*	Standardized canonical coefficients		
					DT	WL	LG
*An. arabiensis*	Females	12, 75	0.90	0.005	1.68	-0.44	-0.06
	Males	12, 75	1.07	0.0004	-1.67	1.13	0.16
*Cx. quinquefasciatus*	Females	12, 75	0.96	0.002	-2.55	0.63	-0.38
	Males	12, 75	1.22	< 0.001	-3.36	0.56	0.15

*Abbreviations*: *df* degrees of freedom, *DT* development time to adulthood, *WL* wing length of adult, *LG* longevity

*Culex quinquefasciatus* females from the control, DAP, and ammonium sulfate treatments took longer to develop compared to those from the glyphosate and cypermethrin treatments (*F*_(4,25)_ = 39.24, *P* < 0.001; Fig. 2b). *Culex quinquefasciatus* females from the DAP and ammonium sulfate treatments were significantly smaller compared to those from the glyphosate and cypermethrin treatments but not from the control (*F*_(4,25)_ = 10.01, *P* < 0.001; Fig. 2d). Females from the control treatments were also significantly smaller than those from the cypermethrin but not the glyphosate treatment (Fig. 2d). There were no significant effects of agrochemical treatment on *Cx. quinquefasciatus* female longevity (*F*_(4,25)_ = 0.77, *P* = 0.55). *Culex quinquefasciatus* male development time was longest in DAP and ammonium sulfate treatments, intermediate in control treatments, and shortest in glyphosate and cypermethrin treatments (*F*_(4,25)_ = 84.99, *P* < 0.001; Fig. 2c). Males from the DAP treatment were significantly smaller than those from the glyphosate and cypermethrin treatments but not from the control and ammonium sulfate treatments (*F*_(4,25)_ = 7.99, *P* < 0.001; Fig. 2e). In addition, males from the control treatment were significantly smaller than those from the cypermethrin treatment (Fig. 2e).

## Discussion

Our results show that agrochemicals can alter the attractiveness and quality of *An. arabiensis* and *Cx. quinquefasciatus* larval habitats. When presented a choice of fertilizer and pesticide-treated oviposition substrates, gravid females of both mosquito species preferentially oviposited in fertilizer-treated substrates. Fertilizer treatments were also associated with higher emergence rates, longer development times, and smaller adults relative to pesticide treatments. These findings are partially consistent with the optimal oviposition theory which predicts that egg laying females should select oviposition sites that maximize the probability for their offspring to reach adulthood and reproduce [53, 54]. To the best of our knowledge, this is the first study to investigate how commonly used agrochemicals affect oviposition site selection and offspring survival of two of the most important mosquito vectors of human pathogens in sub-Saharan Africa.

The impact of fertilizers on mosquito larval populations is well documented. Application of nitrogenous fertilizers in rice fields is often associated with dramatic increase in larval populations of *Anopheles* and *Culex* mosquitoes [9–12, 38]. Similarly, fertilizer-enriched mesocosms and wetlands had higher populations of mosquito larvae compared to control treatments [55, 56]. However, the mechanisms underlying fertilizer-mediated increase in mosquito larval populations are poorly understood. Our results suggest that fertilizer-mediated enhancement of habitat attractiveness and quality may be two of the major factors that account for dramatic increase in mosquito larval populations following application of nitrogenous fertilizers. Fertilizer application promotes microbial growth, which provide chemical cues that aid gravid females to locate suitable oviposition sites [57, 58], stimulate egg hatch [59], and serves as food for mosquito larvae [60],

Avoidance of pesticide-treated oviposition substrates by the two mosquito species was expected as pesticides could be detrimental for egg and larval survival. However, previous studies have documented both positive and negative effects of pesticides on oviposition site selection by mosquitoes. *Aedes aegypti* avoided ovipositing in grass infusion treated with microbial larvicide *Bacillus thuringiensis* var. *israelensis* but did not discriminate between tap water or untreated grass infusion [61]. Gravid females of *Aedes aegypti* were attracted to spinosad-treated oviposition substrates but avoided temephos-treated substrates [62]. Similarly, Eugenol, citronellal, thymol, pulegone, rosemary oil, and cymene acted as oviposition deterrents for *Ae. aegypti* while borneol, camphor and β-pinene acted as oviposition attractants for this mosquito species [63]. Carbaryl-treated pools were more attractive oviposition sites for C*ulex* mosquitoes but had no effect on oviposition behavior of *Anopheles* mosquitoes [31]. Collectively, these findings suggest that pesticides can alter oviposition behavior of mosquitoes but the direction of the response is pesticide-specific. Further studies are needed to establish how a variety of mosquito species respond to different types of commonly used pesticides.

In general, mosquitoes from control and fertilizer treatments took longer to develop and were smaller compared to those from pesticide treatments. Given that pesticide treatments had lower emergence rates compared to control and fertilizer treatments, we believe that random elimination of some larvae by pesticides may have released the survivors from larval competition thereby promoting faster development and larger mosquitoes. It is also possible that pesticides may have selectively favored the survival of larger individuals with rapid growth and development. Both mechanisms have been used to explain why *Aedes* and *Culex* mosquitoes from experimental microcosms exposed to low concentrations of pesticides develop faster and are larger compared to those from control treatments [18, 29, 64]. However, our study design could not allow us to determine which of the two mechanisms was responsible for our observation and further research is needed on this topic.

Mosquito body size is commonly used as a proxy for mosquito fitness and vector potential. Large mosquitoes consume bigger blood meals and lay more eggs compared to small mosquitoes [65]. Large mosquitoes also have longer life spans [2] and are more likely to survive through the extrinsic incubation period of the pathogen compared to smaller mosquitoes [66]. Thus, although fertilizer application may lead to production of large numbers of adult mosquitoes, this may not necessarily translate to increased risk of pathogen transmission since the majority of adults may be small and short-lived. This may be one of the many factors explaining why rice cultivation in East Africa is often associated with large populations of malaria vectors but lower risk of malaria transmission compared to adjacent non-irrigated agroecosystems [67–70]. However, we did not observe any significant effect of agrochemical treatments on adult mosquito life span. Moreover, both fertilizers and pesticides are used simultaneously in many agroecosystems and the large mosquitoes resulting from pesticide treatments may have higher fecundity [64] and longevity both of which may increase the risk of pathogen transmission. However, although exposure of mosquitoes to sublethal concentrations of pesticides is known to enhance arbovirus transmission [28, 29], their impact on malaria and LF transmission is poorly understood. Studies using insecticide-resistant and insecticide-susceptible strains of mosquitoes suggest that exposure to pesticides may reduce the ability of the vector to transmit malaria and LF [71–73] but additional studies are needed to assess how short-term exposure of mosquitoes to sublethal pesticide concentrations affect vector susceptibility to malaria and LF parasites.

## Conclusions

Our results demonstrate that the extensive and widespread use of agricultural chemicals to promote agricultural production can have unexpected consequences on human health by altering epidemiologically relevant mosquito life history traits. In particular, we found that agrochemical treatments can influence where mosquitoes lay eggs, how long they take to complete their development, how many adult mosquitoes are produced, and how big the resulting adults will be. In turn, these traits can influence the spatial and temporal distribution and abundance of mosquito populations and associated pathogens. Additional studies on sublethal effects of agricultural chemicals on mosquitoes and the public health implications are warranted and concerted efforts made to mitigate any potential negative effects of agrochemical use on mosquito-borne diseases.

## Abbreviations

DT, Development time; WL, Wing length; LG, Longevity; df, Degrees of freedom; CTR, Control; AMS, Ammonium sulfate; DAP, Diammonium phosphate; CYP, Cypermethrin; GLY, Glyphosate; ESACIPAC, Eastern and Southern Africa Centre of International Parasite Control; KEMRI, Kenya Medical Research Institute; LF, Lymphatic filariasis; mg/l, Milligrams per liter; MIAD, Mwea Irrigation and Agricultural Development; (NH_4_)_2_HPO_4,_ Diammonium phosphate; (NH_4_)_2_SO_4_, Ammonium sulfate; RH, Relative humidity
